# Genetic contribution to intrinsic functional connectivity underlying general intelligence: evidence from adult twin study

**DOI:** 10.1093/braincomms/fcaf461

**Published:** 2025-11-21

**Authors:** Bishal Guragai, Zhenlan Jin, Toluwani J Amos, Qiuzhu Zhang, Junjun Zhang, Ling Li

**Affiliations:** MOE Key Lab for Neuroinformation, Brain-Computer Interface & Brain-Inspired Intelligence Key Laboratory of Sichuan Province, Center for Psychiatry and Psychology, School of Life Science and Technology, School of Life Science and Technology, University of Electronic Science and Technology of China, Chengdu 610054, China; MOE Key Lab for Neuroinformation, Brain-Computer Interface & Brain-Inspired Intelligence Key Laboratory of Sichuan Province, Center for Psychiatry and Psychology, School of Life Science and Technology, School of Life Science and Technology, University of Electronic Science and Technology of China, Chengdu 610054, China; MOE Key Lab for Neuroinformation, Brain-Computer Interface & Brain-Inspired Intelligence Key Laboratory of Sichuan Province, Center for Psychiatry and Psychology, School of Life Science and Technology, School of Life Science and Technology, University of Electronic Science and Technology of China, Chengdu 610054, China; MOE Key Lab for Neuroinformation, Brain-Computer Interface & Brain-Inspired Intelligence Key Laboratory of Sichuan Province, Center for Psychiatry and Psychology, School of Life Science and Technology, School of Life Science and Technology, University of Electronic Science and Technology of China, Chengdu 610054, China; MOE Key Lab for Neuroinformation, Brain-Computer Interface & Brain-Inspired Intelligence Key Laboratory of Sichuan Province, Center for Psychiatry and Psychology, School of Life Science and Technology, School of Life Science and Technology, University of Electronic Science and Technology of China, Chengdu 610054, China; MOE Key Lab for Neuroinformation, Brain-Computer Interface & Brain-Inspired Intelligence Key Laboratory of Sichuan Province, Center for Psychiatry and Psychology, School of Life Science and Technology, School of Life Science and Technology, University of Electronic Science and Technology of China, Chengdu 610054, China

**Keywords:** resting-state functional magnetic resonance imaging (fMRI), intelligence, functional connectivity, genetic similarity

## Abstract

Resting-state functional connectivity has been linked to intelligence, and twin studies suggest that these associations may be influenced by genetic factors. To investigate this relationship, we analysed behavioural and resting-state functional magnetic resonance imaging data from young adult twins in the Human Connectome Project. General intelligence was assessed based on ten cognitive task performances. The results showed a positive correlation in both identical and fraternal twins, indicating a similarity of general intelligence among twin pairs. For the resting-state functional connectivity analysis, we conducted two approaches. In the first approach, twins were randomly assigned to two separate groups, ensuring that each pair was split between the groups. We then applied a connectome-based predictive method separately for identical and fraternal twins to predict general intelligence. Specifically, a predictive model was trained using one group's functional connectivity and then applied to its co-twin group to predict their general intelligence. Significant prediction was recorded in identical twins but not in fraternal twins, suggesting a high level of similarity of intelligence-related functional connectivity among identical twins. In the second approach, we aimed to quantify the intelligence similarity using the resting-state functional connectivity. To implement this, we generated models to predict the difference in general intelligence in twin pairs, where a smaller difference indicates a greater degree of similarity. The results showed that only the intelligence difference in identical twins was successfully predicted, where the default mode network showed a significant contribution, suggesting a higher neural basis for intelligence similarity in identical twins. Together, these findings demonstrate that functional connectivity patterns associated with intelligence extend across genetically identical twins. More broadly, they highlight the default mode network role in intelligence similarity and illustrate the utility of predictive modelling as a complementary framework to classical twin analyses.

## Teaser

Guragai *et al*. report that identical twins show stronger similarity in brain connectivity linked to intelligence than fraternal twins. Using resting-state functional imaging, they demonstrate that genetic factors shape intelligence-related connectivity, with the default mode network playing a key role in mediating this similarity.

## Graphical Abstract

**Graphical Abstract:**
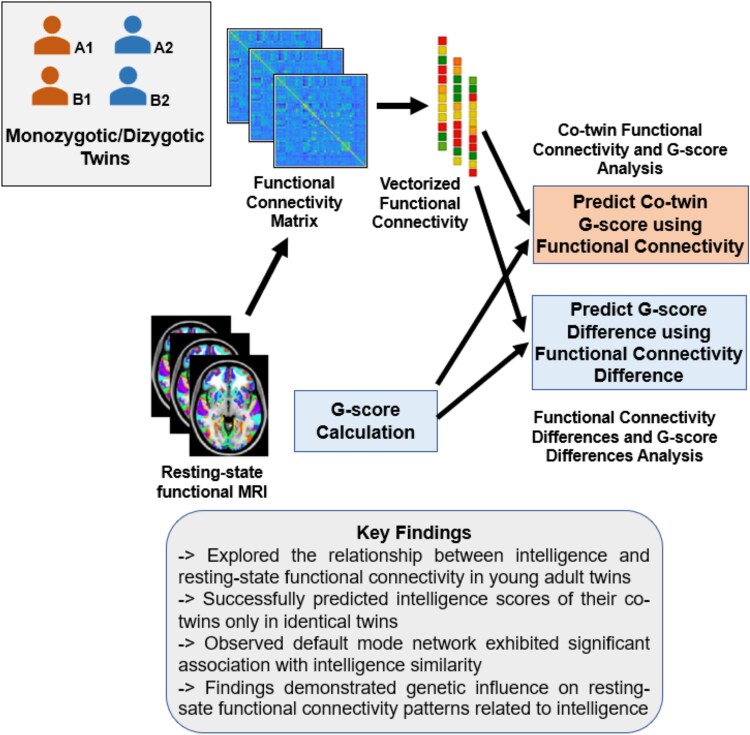

## Introduction

Intelligence is a complex and diverse cognitive ability, including reasoning, problem-solving, memory and the capacity to learn from experience.^1-3^ The structure of intelligence is composed of numerous distinct but related constructs. Early psychometric theories, like Spearman's proposed general intelligence ‘g’ factor, are believed to account for diverse cognitive abilities.^1^ The general intelligence accounts for around half of the variation in intellectual abilities^4^ and demonstrates good reliability when applied to various cognitive tasks.^5^ The empirical observation of general intelligence has been firmly established, but its interpretation has been challenged and remains an actively debated topic in the field.^6-8^

Resting-state functional connectivity (rsFC) has been highlighted to be unique and stable for an individual over extended periods, even across months to years.^9,10^ The consistency of rsFC patterns has enabled its use as an effective tool for identifying individual differences in brain organization.^11-15^ Among the methods leveraging rsFC, the connectome-based predictive modelling (CPM) method, proposed by Shen,^16^ has been widely adopted to develop neuroimaging-based biomarkers and successfully predict individual differences. Studies employing CPM have demonstrated that rsFC patterns predict differences in cognitive abilities, including attention,^17^ general intelligence,^18^ intelligence quotient (IQ),^19^ fluid intelligence (Gf),^11^ working memory,^20^ and creativity.^21^ Specifically, the default mode network (DMN), and frontoparietal network (FPN) showed a strong association with intelligence.^18,19,22^ Moreover, the middle frontal gyrus significantly contributes to predicting fluid and crystallized intelligence when analysed using graph neural networks applied to resting-state connectivity,^23^ further underscoring the link between rsFC and cognitive traits. Beyond functional connectivity, studies have mapped spatial relationships between in vivo rsFC patterns and ex vivo gene expression from post-mortem tissue, revealing genetic influences on rsFC.^24,25^ Resting-state networks also exhibit high heritability, suggesting genetic factors shape intrinsic functional architecture.^26-28^ Collectively, these findings imply a genetic contribution to rsFC patterns associated with intelligence, though the precise mechanisms remain unclear.

Twin studies provide an effective methodological framework for estimating the contribution of genetic factors to intelligence. Monozygotic(identical) twins are genetically more similar compared to dizygotic(fraternal) twins, who share approximately 50% of their segregating genes on average.^29-31^ Genetic variations significantly influence the formation of the rsFC across brain regions, which in turn affects cognitive abilities.^32,33^ Twin-based studies have demonstrated a significant correlation in general intelligence between co-twins, suggesting that individual differences in intelligence are partly attributable to genetic factor.^34-36^ At the neural level, monozygotic twins show higher whole-brain rsFC similarity compared to dizygotic twins.^37^ Furthermore, rsFC patterns exhibit remarkable stability from youth to adulthood, with this similarity scaling according to genetic relatedness.^27,28^ Fu and colleagues (2015) advanced this understanding by demonstrating how genetic factors shape individual resting-state networks, proposing that synchronized spontaneous neural activity reflects the brain’s intrinsic organization.^26^ Studies found a significant genetic correlation in intelligence-related networks such as DMN, ventral attention and salience networks during the resting state, suggesting shared genetic influences across these networks.^33,38^ Notably, intelligence-associated networks—including the DMN, ventral attention network, and salience network—show significant genetic correlations during rest, indicating shared genetic influences. Emerging evidences further suggest that spatial patterns of gene expression align with these large-scale brain connectivity networks, supporting their role in cognition.^39,40^ Collectively, these findings suggest that rsFC patterns associated with intelligence are more similar in twins with higher genetic relatedness. However, the extent to which genetic similarity specifically shapes intrinsic connectivity patterns underlying intelligence remains unexplored.

This study aimed to investigate whether intrinsic functional connectivity patterns underlying general intelligence generalize across twins who differ in genetic similarity. Using resting-state fMRI data from identical (MZ) and fraternal (DZ) twin pairs in the Human Connectome Project, we examined the similarity of intelligence-related connectivity profiles across twins. Our analysis employed a two-pronged approach: First, we applied a connectome-based predictive framework to test whether a twin’s rsFC patterns predict their co-twin’s intelligence. We further validated whether intelligence-related rsFC patterns can accurately identify a twin’s co-twin from among all other individuals in the cohort. Second, we quantified intelligence similarity by generating rsFC-based models to predict differences in intelligence similarity between twins, where a larger score difference signifies lower similarity. Our results showed two key findings: First, the successful prediction of a co-twin’s intelligence only in identical twins, indicating higher rsFC patterns similarity in genetically identical twins. Second, the degree of intelligence similarity can predict rsFC patterns in identical twins, with the DMN playing a particularly important role. By combining a predictive modelling framework with a twin design, the current study offers a complementary approach to classical genetic analyses and sheds light on how genetic factors contribute to the connectivity patterns underlying human intelligence.

## Methods

### Neuroimaging dataset

In this study, we utilized a dataset of young and healthy adult twins obtained from the Human Connectome Project (HCP) (https://www.humanconnectome.org) 1200-subject release. A special authorization was obtained to use both the Open and Restricted Access data from the HCP. There were 184 twin pairs (MZ = 120 pairs, DZ = 64 pairs, age: 22–36 years) in the 1200-subject release of the HCP dataset, and the subjects who met the following criteria were retained for the analyses: (i) subjects who completed all relevant neuropsychological tests (NEO-FFI_Compl = True, SCPT_Compl = True, PMAT_Compl = True, Non-TB_Compl = True, VSPLOT_Compl = True, VisProc_Compl = True and IWRD_Compl = True) and the Mini Mental Status Exam (MMSE_Compl = True);^18^ (ii) subjects who had all four rs-fMRI runs (3T_RS-fMRI_PctCompl = 100); and (iii) subjects whose estimated framewise root-mean-square motion in any of the four rs-fMRI runs did not exceed 0.2 mm.^41^ Lastly, only if both twins met the above criteria, were their twin pairs included in the analysis. As a result, 139 same-gender twin pairs (89 pairs of MZ, 51 female pairs; 50 pairs of DZ, 30 female pairs; Supplementary Table 1) remained for the analysis.

The zygosity of the twins has been genomically validated as of March 2017, and twin status was determined based on this genomic verification from blood or saliva samples. These data can be accessed through the database of genotypes and phenotypes (dbGaP) maintained by the National Center for Biotechnology Information (NCBI) (https://www.ncbi.nlm.nih.gov/projects/gap/cgi-bin/study.cgi?study_id=p hs001364.v1.p1). The collection of these data adhered to the principles outlined in the Helsinki Declaration and was authorized by the Washington University Institutional Review Board (IRB #201204036).^42^

### Assessment of general intelligence

We calculated general intelligence scores (G-scores) using the bifactor modelling framework developed by Dubois *et al*. (2018).^18^ This approach simultaneously estimates both a general cognitive factor (g) and four specific cognitive domain factors through exploratory factor analysis. To assess the G-score, we selected 10 cognitive measures from the database of HCP, sourced from the NIH Toolbox (NIHTB) (https://www.nihtoolbox.org/domain/cognition^43^) and the Penn Computerized Neurocognitive Battery (CNB).^44^ The model was fitted using exploratory factor analysis with a bifactor model in the Lavaan package of version 0.6.5^45^ within the R-studio Software of version 3.6.1 for statistical computing. Model fit was measured using the comparative fit index (CFI), the test of χ^2^-goodness, the standardized root-mean-square residual (SRMR), and the root–mean-squared error of approximation (RMSEA). An acceptable model should have a value of CFI greater than 0.95, whereas both the RMSEA and SRMR values should be less than 0.08.^46^ The final factor loadings for both general and specific factors are presented in Supplementary Fig. 1. For further details regarding cognitive measures, see the descriptions in the Data Dictionary’s Cognition Category provided by HCP (https://wiki.humanconnectome.org/docs/HCPYA%20Data%20Dictionary-%20Updated%20for%20the%201200%20Subject%20Release.html). To accurately identify each cognitive task measure in the obtained model, we utilized age-unadjusted scores for all measures and the initial abbreviated task names provided by the HCP dataset. The four specialized factors with their cognitive measures are (i) cry, i.e. crystallized intelligence (PicVocab_Unadj and ReadEng_Unadj); (ii) vis, i.e. visuospatial ability (PMAT24_A_CR and VSPLOT_TC); (iii) spd, i.e. processing speed (ProcSpeed_Unadj, CardSort_Unadj and Flanker_Unadj); and (iv) mem, i.e. memory (ListSort_Unadj, IWRD_TOT and PicSeq_Unadj).

G-score was adjusted for age and gender to account for their potential confounding effects on the intelligence measure.^18,47-49^ Consistent with these previous approaches, we employed multiple linear regression to control for these demographic variables in our dataset (see Supplementary Table 1 for detailed information). The resulting age- and gender-adjusted G-scores were subsequently used in all analyses.

### Neuroimaging acquisition and preprocessing

The rs-fMRI data were acquired using a gradient-echo echo planar imaging (EPI) sequence of (echo time (TE) = 33.1 ms; repetition time (TR) = 720 ms; slices = 72, voxel size = 2 × 2 × 2 $mm^{3}$; field-of-view (FOV) = 208 × 180 $mm^{2}$; volumes = 1200) from a Siemens Skyra 3 Tesla scanner. The acquisition parameters applied to the rs-fMRI data are comprehensively described in the original article.^50^ During the resting-state scan, the participants were instructed to look at the fixation cross with their eyes open. Briefly, the rs-fMRI acquisition protocol involved two sessions on different days, with each session having two separate 14 min 24 s scans. Each session includes rs-fMRI phase encoding data from left-right and right-left directions, resulting in four runs per participant. The present study used the preprocessed data, including the following preprocessing steps: (i) EPI geometric distortions and head motion movements were rectified, and were registered to the standard template of the T1-weighted image and then normalized to the MNI152 template.^50,51^ (ii) independent component analysis-based X-noiseifier (ICA-FIX) was used to eliminate noise artefacts linked to intense head movement, and pulsations from cardiac, arteries and vein-related effects.^52^ On these preprocessed data, we further used a band-pass filter at 0.009–0.08 Hz to reduce the influence of low- and high-frequency noises.^53^ In addition, we regressed out the global mean signal, the white matter volume, and the cerebrospinal fluid (CSF) as confounding variables utilizing Data Processing and Analysis for Brain Imaging (DPABI) MATLAB toolbox (http://www.rfmri.org/dpabi).^54^

### Estimation of vectorized functional connectivity

**Figure 1:**
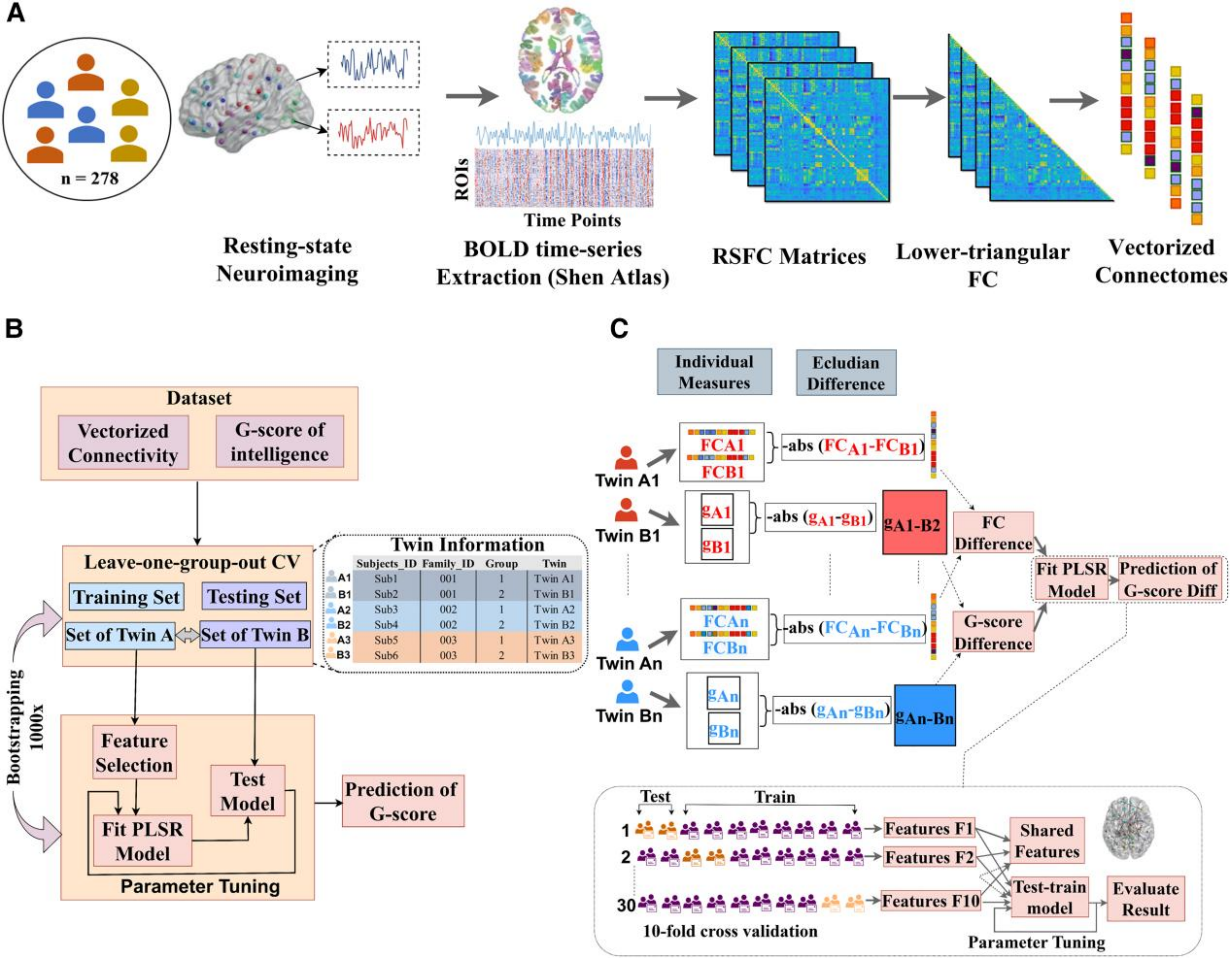
**Overview of our study**. (**A**) Construction of one-dimensional vectorized functional connectivity from rs-fMRI data across 278 subjects (89 MZ twin pairs and 50 DZ twin pairs). (**B**) Workflow for predicting the G-score of twins based on their co-twin data using partial least squares regression with a leave-one-group-out cross-validation (LOGOCV) scheme. (**C**) Workflow for assessing the association between functional connectivity differences and G-score differences using 10-fold cross-validation (rsFC: resting-state functional connectivity; CV: cross-validation; PLSR: partial-least square regression).

We used Shen’s parcellation atlas containing 268 regions of interest (ROIs)^55^ to characterize whole-brain resting-state functional connectivity. For each resting-state run, the average blood oxygen level-dependent (BOLD) time series for each region of interest was extracted for each subject. Subsequently, pairwise Pearson correlation coefficients were computed between all 268 ROIs, resulting in a symmetric 268 × 268 functional connectivity matrix representing edges (ROI-to-ROI connections). We then used a Fisher transformation to normalize the matrix to *z*-scores. To generate the average functional connectivity matrix for each subject, we averaged the Fisher transformation matrix of four runs. From the averaged symmetric matrix with dimensions 268 × 268, only the lower triangular part (excluding the diagonal components, which represent the correlation of each ROI with itself) was used to generate a one-dimensional vectorized representation of the functional connectivity (Fig. 1A). The resulting 1-dimensional vector for each subject containing 35 778 unique edge weights represented the whole-brain intrinsic connectivity of that subject, also called vectorized functional connectivity.

### Association between the G-score and the functional connectivity of twins

#### Predicting G-score with the model generated using co-twins functional connectivity

We first investigated whether individual differences in G-score could be predicted from rsFC patterns. The analysis was aimed at determining whether intrinsic functional connectivity patterns can serve as a significant predictor of an individual’s G-score. We used partial-least square regression (PLSR) with a leave-one-out cross-validation approach. PLSR was chosen for its efficacy in handling high-dimensional and collinear datasets,^56,57^ as it maximizes covariance between predictors (i.e. functional connections) and the response variable (i.e. G-score) through a dimension reduction approach. To validate our findings, we also explored additional predictive algorithms, including support vector machines (SVM) regression (radial basis function (RBF) kernel), decision trees regression, and logistic regression, and compared their performances. Model performance was quantified using Pearson correlation coefficients between predicted and observed G-scores in the held-out test sets. Then, we calculated *P*-values by comparing the mean correlation coefficient value obtained from the folds with a null distribution of coefficient values generated through permutation testing.

We used a leave-one-group-out cross-validation (LOGOCV) approach (Fig. 1B) to investigate the similarity of rsFC patterns in twins. The analysis involved randomly partitioning twin pairs into two sets, a training set and a testing set. For example, if A and B are twins, then twin A and twin B were grouped into the training set and testing set, respectively. Using the training set, we developed a model to predict G-scores, which were subsequently evaluated on the independent testing set. We used the Scikit-learn function ‘f_regression’, a feature selection method, which identified the most strongly associated features based on F-statistics (*P* < 0.05). We optimized model parameters through grid search across PLSR components ranging from 1 to 100. To evaluate the model performance, we calculated the Pearson correlation between actual and predicted G-scores.

To mitigate the impact of the arbitrary grouping of twins into the training and testing sets, we implemented a bootstrapping procedure with 1000 iterations. For example, in one iteration, the training set may include data labelled with identifier ‘A’ (e.g. 1A, 2A, 3A and so on), while the testing set may include data labelled with identifier ‘B’ (e.g. 1B, 2B, 3B and so on). In another iteration, one or more twin pairs may swap between the training and testing sets (e.g. 1A, 2B, 3B and so on for the training set, and e.g. 2B, 2A, 3A and so on for the testing set). Every sample was either present in the training or testing set, but never in both sets. This grouping procedure was repeated 1000 times, resulting in 1000 predictive models.

We performed 100 randomized permutations for each of the 1000 predictive models, generating a null distribution comprising 100 000 chance-level performance coefficients. The average coefficient value from 1000 models was subsequently compared to a null distribution of the coefficient values generated through permutation testing.

Briefly, in this approach, the predictive model was trained on a group of twins’ data (Twin A), and then was tested on the connectivity data of another group of twins (Twin B) to predict their G-scores in this approach.

#### Functional connectivity contributing to the predictive models

We further identified functional connections (features) that were consistently selected across folds (two folds for LOGOCV) in the one-thousand bootstrapped models while predicting the G-scores of co-twins. We further demonstrated the selected connections by identifying those that appeared in more than 50% of the bootstrapped models (i.e. 500 out of 1000). We then computed the node degrees to which brain regions are interconnected and how strongly they communicate with each other. The node degree was computed by summing up the connections to that node, named connectivity strength (for reference, see BioImage Suite Connectivity Viewer (bioimagesuiteweb.github.io). Higher connectivity strength indicates more communication and interaction with other brain regions.^16^ We obtained the top five nodes for their outstanding contribution to the across-group prediction and where these nodes belonged.

#### Validation of co-twin identification using functional connectivity

We evaluated whether intelligence-related FC patterns (those consistently selected across folds during the cross-validated prediction of G-scores) can accurately identify a twin’s co-twin from among all other individuals in the cohort. Specifically, for a given twin attempting to identify their co-twin, we computed the Pearson correlation coefficients between their intelligence-related FC patterns and those of every other individual in the cohort. This process generated a set of Pearson correlation coefficients for each twin. The individual with the highest correlation coefficient was selected as the index of identification. We then calculated the identification accuracy as the percentage of cases where the true co-twin was correctly identified. This analysis was performed separately for MZ and DZ twin cohorts. Using the same methodology, we also performed co-twin identification based on whole-brain FC patterns. The identification performance using whole-brain FC patterns was then compared to that achieved using intelligence-related FC patterns.

### Association between the G-score difference and the functional connectivity difference of twins

#### Predicting the G-score difference of twins using the functional connectivity difference

To quantify the intelligence similarity between twins, we generated predictive models of the G-score differences. First, we computed Euclidean distance (i.e. absolute values of numerical difference) for both the G-score and vectorized rsFC patterns of within twin pairs (Fig. 1C), where larger G-score differences reflected lower intelligence similarity. We then used a regression model with a ten-fold cross-validation to predict these G-score differences from rsFC differences. The dataset was randomly divided into 10 equal folds, with models trained on 9 folds and tested on the remaining fold. We used the Scikit-learn function f_regression, feature selection method, retaining the top 400 most significant features for regression analysis. PLSR parameters were optimized through grid search across components ranging from 1 to 100. Then, the Pearson correlation coefficient was computed between the actual G-score difference and predicted G-score difference to evaluate the ability of model's performance, with statistical significance assessed through 5000 iterations of permutation testing. To validate our approach, we further explored additional predictive algorithms, including SVM (RBF kernel), decision trees and logistic regression, applying identical cross-validation and feature selection procedures across all models to ensure consistent comparison.

#### Functional connectivity contributing to G-score differences

Building upon our predictive modelling approach, we identified rsFC patterns contributing to G-score differences by examining connections consistently selected across all 10 cross-validation folds. Based on the selected connections, we focused on the top five nodes demonstrating the strongest connectivity. The connectivity strength of a node was calculated as the sum of all weighted connections to each node. To present our result in network-level perspective, we then we mapped the 268 nodes onto 10 canonical functional networks (Finn *et al*., 2015; Noble *et al*., 2017): medial frontal (MFN; 29 nodes), frontoparietal (FPN; 28), default mode (DMN; 18), motor (MOT; 49), visual I (VISI; 18), visual II (VISII; 9), visual association (VAN; 18), limbic (LMB; 30), basal ganglia (BG; 29) and cerebellum (CRB; 40).

Following the methodology, we evaluated each network's predictive capacity by constructing separate models for G-score difference prediction. For each network, we extracted intra-network functional connectivity values from the 268 × 268 FC matrix, generating one-dimensional vectorized rsFC representations. This network-specific approach allowed us to quantify the relative contribution of distinct functional systems to intelligence similarity between twins.

### Statistical validation of predictions

For the statistical comparison of correlations from identical twins and fraternal twins, we used the ‘Cocor’ package in R for the statistical comparison of correlations from independent groups.^58^ The ‘Cocor’ package also incorporates the application of Zou’s confidence interval implementation for comparisons.^59^ To ensure sufficient statistical power, we used G*Power 3.1 (Erdfelder *et al*., 2009) to calculate the power to detect expected differences in correlation coefficients, based on the observed effect sizes and the sample sizes in our study.

## Results

The final analysis included 139 twin pairs (89 pairs of MZ twins and 50 pairs of DZ twins) that met all the inclusion criteria. We derived general intelligence scores (G-scores) from 10 cognitive measures, then adjusted these for age and gender effects due to their significant correlations with unadjusted scores (see detailed information in Supplementary Table 2). These adjusted G-scores served as our primary measure of general intelligence in subsequent analyses. In both MZ and DZ twins, the G-scores in twins were significantly correlated, with a numerically higher correlation observed in MZ twins (MZ: *r* = 0.78, *P* = 3.1 × $10^{-6}$; DZ: *r* = 0.42, *P* = 4.5 × $10^{-4}$). This finding suggests a greater similarity in G-scores within MZ twins compared to DZ twins, consistent with a genetic influence on general intelligence. Preliminary heritability estimates using Falconer’s formula ($h^{2}=2\;*\;(r_{MZ}-r_{DZ})$) indicate that approximately 72% of the variance in G-scores may be attributable to genetic factors.

### Predicting G-score using their co-twins’ functional connectivity

We observed a significant correlation between resting-state functional connectivity and G-scores in both MZ and DZ twin cohorts (MZ: *r* = 0.36, *P* = 2.3 × $10^{-5}$, confidence interval = [0.2, 0.52]; DZ: *r* = 0.23, *P* = 1.6 × $10^{-4}$, confidence interval = [0.08, 0.38]). These results suggest that individual differences in intrinsic functional brain organization are related to variations in intelligence.

**Figure 2:**
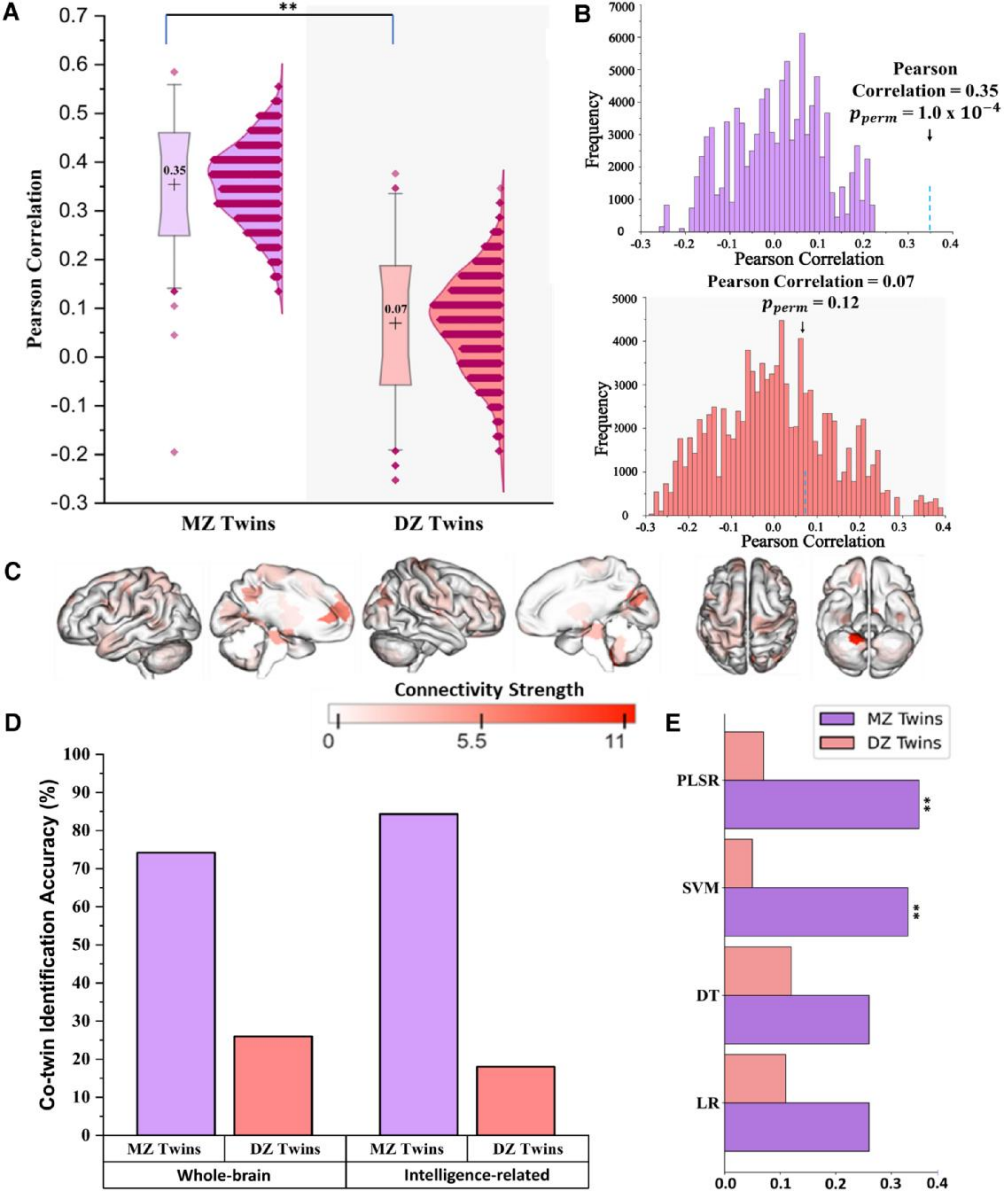
**Association of functional connectivity with G-score**. (**A**) Box plots depict the mean, quartiles and potential outliers of the distribution. Distribution plots provide a detailed view of the frequency distribution for different correlation values when predicting co-twin G-scores. The grey-shaded area represents the distribution of DZ twins, which lacks statistical significance in predicting co-twin G-scores. The mean correlation coefficients were higher in MZ twins (*N* = 89) than DZ twins (*N* = 50) (*t* = 65.23, **P < 0.01, two-tailed *t*-test). (**B**) Histograms show the null distribution, with a blue vertical dotted line indicating the mean score for both MZ (top) and DZ (bottom) twins. (**C**) Neural bases contributing to G-scores that were consistently selected across two folds in MZ twins (no. of edges = 79). (**D**) Bar graph depicting co-twin identification accuracy (%) using whole-brain and intelligence-related functional connectivity (FC) patterns in MZ and DZ twins. (**E**) Predictive performance of different regression models in estimating individual G-scores from co-twin functional connectivity. Bars represent the mean correlation coefficient (r) across 1000 iterations. Asterisks (**) denote models that survived the permutation test (PLSR: mean *r* = 0.35, **p_perm < 0.01, SVM: mean *r* = 0.33, **p_perm < 0.01). (MZ: monozygotic; DZ: dizygotic; LR: logistic regression; DT: decision tree; SVM: support vector machine; PLSR: partial-least square regression; p_perm: permutated p-value).

Importantly, the LOGOCV method, which was applied for each separation of twins into two groups (1000 times of random separation of twins into two groups), revealed the predictive model trained on a group of twin data predict their co-twins G-score. The model trained on MZ twins successfully predicted their co-twins’ G-scores (mean *r* = 0.35, confidence interval = [0.24, 0.45]), while DZ twins (mean *r* = 0.07, confidence interval = [−0.05, 0.19]) (Fig. 2A). The predictability in MZ twins survived the permutation test (iterations = 100 000; $p_{\;perm}$ = 1.0 × $10^{-4}$, Fig. 2B top), but not in DZ twins (iterations = 100 000; $p_{\;perm}$ = 0.12, Fig. 2B bottom). Furthermore, we also compared the mean correlation coefficients obtained from the MZ and DZ twins using a *t*-test and found a significant difference (*t* = 65.23; *P* = 1.1 × $10^{-12}$; power = 1), indicating higher predictability of co-twin’s G-score using rsFC in MZ twins. Similarly, comparison test using Cocor’s package and G*power also revealed the predictability of the MZ twins was significantly higher than DZ twins (Fisher’s *z* = 2.33, *P* = 0.019, confidence interval = [0.044, 0.513], two-tailed). We further validated these findings using alternative predictive algorithms including SVM, decision trees and logistic regression, and compared their performances. Among these, the PLSR algorithm demonstrated superior performance in terms of prediction accuracy. While both decision trees and logistic regression failed to achieve statistical significance (Fig. 2E, also see Supplementary Table 5).

Since only the MZ twins showed significant G-score predictability using the model trained on their co-twin’s functional connectivity patterns, we examined the features that appeared more than 50% in the one-thousand predictive models of the MZ twins. We obtained seventy-nine edges that were mostly from the medial-frontal network (MFN), VISI network and CRB (Fig. 2C). The top five nodes that had high connectivity strength comprised the cerebellum (11), left anterior prefrontal cortex (6), left visual cortex (6), right visual cortex (6) and right insular cortex (5). These findings highlight specific intrinsic functional connectivity patterns that may underlie the prediction of intelligence in MZ twins (Table 1).

**Table 1:** Top five brain regions consistently selected in MZ twins exceeding 50% threshold

Node Name	Broadmann Areas	Network	MNI coordinates (*X*, *Y*, *Z)*	Connectivity Strength
Right Cerebellum	Cerebellum	Cerebellum	(16.18, −47.2, −52.31)	11
Left Visual Cortex	PrimVisual (BA17)	Visual I	(22.11, −66.7, 7.45)	6
Left Anterior Prefrontal Cortex	AntPFC(BA10)	Medial-Frontal	(−5.96, 48.09, 11.72)	6
Right Visual Cortex	SecVisual (BA18)	Visual I	(7.71, −74.97, 25.03)	6
Right Insular cortex	Insula (BA13)	Motor	(38.34, −12.45, −1.09)	5

*MNI, Montreal Neurological Institute*.

Furthermore, we performed a co-twin identification analysis on functional connectivity patterns to further validate our findings. The identification accuracy was quantified as the percentage of cases where the true co-twin was correctly identified. For MZ twin pairs using intelligence-related FC patterns (those consistently selected across folds during the cross-validated prediction of G-scores), we achieved significantly higher identification accuracy for MZ twins (82.0%) compared to DZ twins (28.9%). When employing whole-brain FC patterns, the accuracy was 74.2% for MZ twins and 26% for DZ twins (Fig. 2D). These findings suggest that intelligence-related FC patterns show a higher genetic contribution compared to the overall FC patterns, particularly in MZ twin pairs.

### Predicting G-score difference using functional connectivity difference in twin pairs

**Figure 3:**
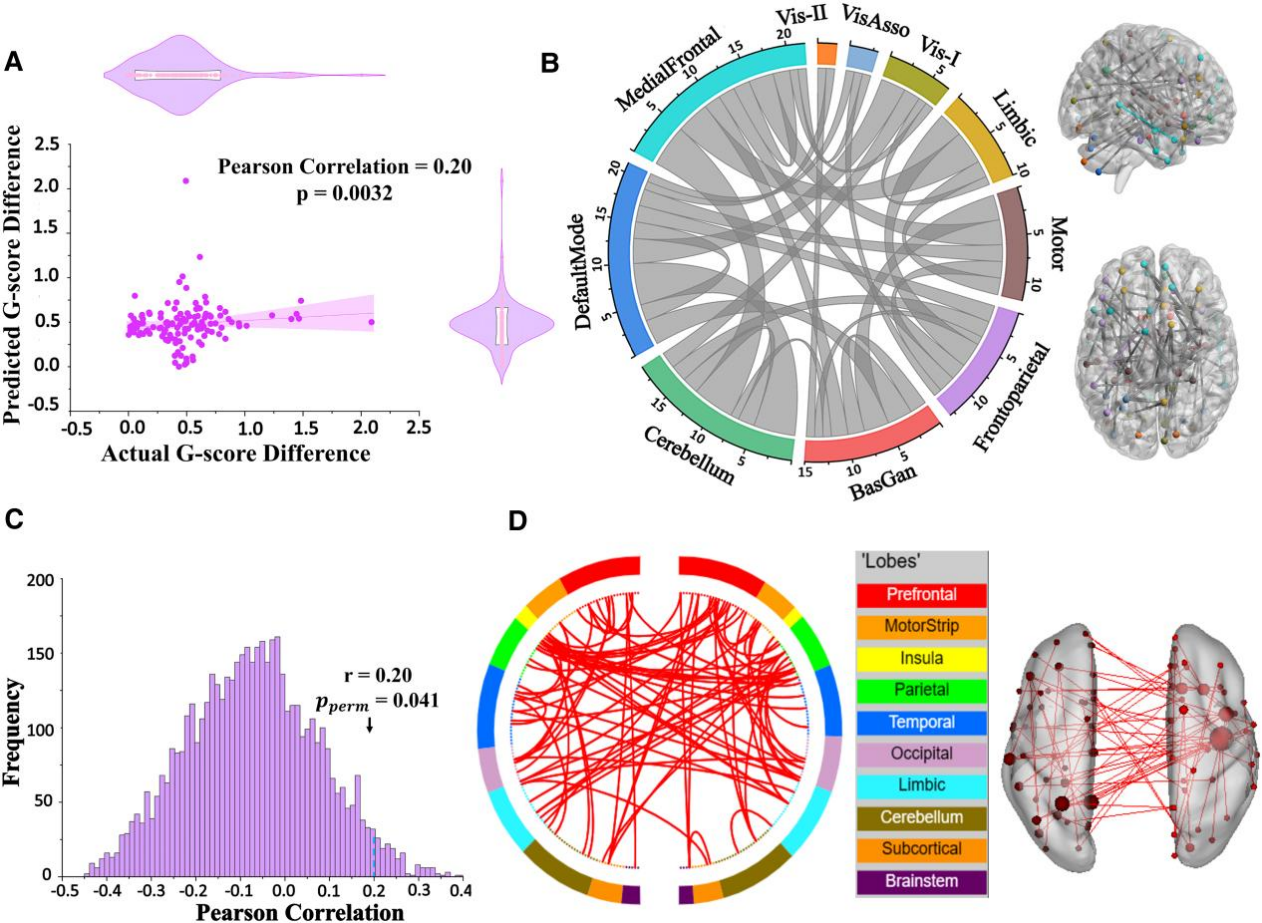
**Association of G-score differences and functional connectivity differences in MZ twins**. (**A**) Functional connectivity differences predicted G-score differences in MZ twins. The accompanying violin plot (embedded with a box plot) displays the distribution of both actual and predicted scores (Pearson Correlation, *r* = 0.2, *N* = 89). (**B**) Chord diagram illustrating connections within and between brain networks. (**C**) Histogram showing the null distribution of G-score differences, with a line indicating the observed true score. (**D**) Chord diagram highlighting interhemispheric connectivity between right and left hemispheres across Shen’s brain lobes. (*P*: p-value without permutation; p_perm: permutated p-value; r: Pearson correlation; Vis-I: visual I; Vis-II: Visual II; VisAsso: visual association; BasGan: basal ganglia).

Our analysis using 400 selected features revealed significant predictability of G-score differences from functional connectivity (FC) differences in MZ twins (*r* = 0.20, *P* = 0.0032, Fig. 3A) with results surviving permutation testing (iterations = 5000, $p_{\;perm}$ = 0.041, Fig. 3C). In contrast, DZ twins showed no significant predictability (*r* = −0.07, *P* = 0.32; iterations = 5000, $p_{\;perm}$ = 0.11; Supplementary Fig. 2A and 2B), suggesting that FC differences cannot reliably predict intelligence differences. However, direct comparison of the correlation coefficient in MZ twins using the ‘Cocor’ package showed that no statistically significant difference (Fisher’s *z* = 1.50, *P* = 0.13, two-tailed; power 0.324, effect size *q* = 0.2728). To further characterize this relationship, we systematically varied the number of features included in the predictive model, demonstrating how feature set size affects prediction accuracy (Supplementary Fig. 3).

Upon the predictability of G-score difference in the MZ twins, we found the right parietal angular gyrus (BA39) and right inferior frontal gyrus as the most influential nodes, each demonstrating a connectivity strength of 6 (Fig. 3D; detailed information in Supplementary Table 3). At the network level, the medial-frontal network (MFN), DMN, and CRB were most frequently selected across cross-validation folds, while the visual regions (VISI, VISII and VAN) exhibited a lower degree of selection (Fig. 3B). We further analysed individual networks’ predictive capacity. Notably, only the DMN significantly predicted the G-score differences in MZ twins (*r* = 0.25, *P* = 0.009; iterations = 5000, $p_{\;perm}$ = 0.012), with no significant prediction observed in DZ twins (*r* = −0.12, *P* = 0.13; iterations = 5000, $p_{\;perm}$ = 0.32) (Supplementary Table 4). However, direct comparison using Cocor’s package confirmed significantly higher predictability in MZ twins (Fisher’s *z* = 2.07, *P* = 0.038, two-tailed; Confidence Interval, [0.02, 0.69]; power 0.545, effect size *q* = 0.3760), underscoring the crucial role of the DMN in intelligence similarity. For validation, we compared these results with support vector machine (SVM) predictions, excluding decision tree and logistic regression approaches due to their previously demonstrated non-significant results. Comparatively, the PLSR algorithm demonstrated superior performance in terms of prediction accuracy (see Supplementary Table 6).

## Discussion

In the present study, we investigated genetic contributions to intrinsic functional connectivity patterns associated with general intelligence (G-score) in young adult twins from the Human Connectome Project (HCP). We focused on identical (MZ) and fraternal (DZ) twin pairs to test whether brain–behaviour associations generalize across individuals who differ in genetic similarity. By adopting a cross-twin predictive framework, we tested whether models trained on one co-twin’s connectivity could predict the other co-twin’s intelligence, providing a direct assessment of out-of-sample generalization within families. Our findings revealed that similarity in intelligence-related functional connectivity was aligned with genetic relatedness, with four key observations emerging. First, only identical twins showed significant predictability of G-scores using models trained on co-twin connectivity patterns. Second, we observed significantly higher identification accuracy for identical twin pairs using intelligence-related functional connectivity patterns (selected across folds during the cross-validated prediction). Third, brain regions from the MFN, Visual-I network and cerebellum primary contributors to the prediction of co-twin’s G-scores, suggesting these regions represent genetically influenced substrates for intelligence. These intrinsic connectivity networks provide a mechanistic framework that can elucidate how the brain organizes to process information relevant to cognition.^60,61^ Third, the DMN played a specialized role, with its connectivity differences specifically predicting G-score differences in identical twins. These findings collectively demonstrate that functional connectivity patterns predictive of general intelligence in one twin generalize to their co-twin in identical pairs, consistent with genetic influences on cognition-relevant connectivity, while also highlighting the utility of predictive modelling as a complementary framework to classical twin analyses. By leveraging genetically informative samples, our approach advances understanding of how genetic factors contribute to the brain networks supporting intelligence.

In the present study, we found significant behavioural similarity in general intelligence (G-scores derived from 10 cognitive tasks) between both identical and fraternal twins, consistent with established genetic contributions to cognitive abilities.^62,63^ This aligns with evidence that certain genetic loci combining the effects of multiple gene variations are significantly associated with intelligence and can explain more than 50% of variance in an individual’s intelligence measures^64^ and that individual differences are reflected in functional brain architecture.^18,19,65^ Here, we used the LOGOCV approach. There are several multivariate connectivity-based predictive methods, such as CPM,^16^ and support vector regression (SVR),^66^ to predict individual differences of intelligence from functional connectivity patterns Still, the LOGOCV method opened up a novel avenue for predicting intelligence from functional connectivity patterns. Using the method, we found that models trained on identical co-twin connectivity successfully predicted G-scores—an effect absent in fraternal twins. This extends previous findings that identical twins exhibit more similar functional connectivity patterns than fraternal twins,^27,28^ by specifically linking genetic factors to intelligence-related connectivity patterns. Our results support a model whereby genetic influences shape intrinsic connectivity, particularly in hub regions critical for high-level information processing.^67,68^ Moreover, these genetic influences may be mediated by spatial patterns of gene expression,^69^ affecting neural circuits through genes involved in neuron differentiation,^70^ and may ultimately contribute to individual differences in intelligence.^33,71^ Thus, our approach provides a framework for evaluating the biological processes underlying intelligence by identifying the specific connectivity patterns that meditate genetic influences on intelligence.

We observed that the most influential functional connections for co-twin intelligence prediction with high connectivity strengths were the cerebellum, left anterior prefrontal cortex, bilateral visual cortex and right insular cortex. These nodes are primarily localized to the medial-frontal network (MFN), VISI network and CRB. Studies have emphasized the importance of the medial-frontal cortex in influencing cognitive abilities assessed by various neuropsychological tests.^72,73^ Specifically, the medial orbitofrontal (MOF) subregions have been found to significantly contribute to individual differences in general intelligence.^73^ The highly structured pathways and well-aligned axonal tracts within the MOF may facilitate efficient neural communication, thereby enhancing intelligence. Similarly, the significant contribution of the visual-I network may reflect its strong genetic determination, as visual system development occurs early and shows relatively less driven by environmental factors, but more by genetics.^74^ For cerebellar involvement, we propose two potential explanations. First, the cerebellum is highly susceptible to environmental changes. A previous study demonstrated that environmental enrichment can enhance cerebellar compensation and promote the development of cerebellar reserve, significantly impacting the structure and function of the cerebellum.^75^ Though our study did not directly assess environmental factors. Second, the observed patterns may have been impacted by the resolution and complexity of the parcellation.

Our second analytical approach quantified intelligence similarity between twins using functional connectivity differences. Here, we computed the G-score difference in twin pairs to reflect the intelligence similarity, where a higher difference means less similarity. At first, we found functional connectivity differences significantly predicted G-score differences in identical twins, but not in fraternal twins. However, when directly comparing the predictability of models, we did not find any significant differences in both identical and fraternal twins. Considering the differential roles of brain networks, we performed network-specific analyses, then found that only the DMN predicts intelligence differences in identical twins, with significantly greater predictive accuracy compared to fraternal twins. Our results highlighted a critical role of the DMN in quantifying intelligence similarity in identical twins, and in line with previous findings that genetic influences on DMN connectivity.^76-78^ The DMN is involved in cognitive processes such as thinking and comprehension, and undergoes significant growth and development throughout adolescence and into early adulthood.^79,80^

While our findings provide insights into genetic influences on intelligence-related functional connectivity, there are some limitations. First, our approach explores the effectiveness of multivariate pattern similarity analysis in understanding functional specialization, as well as individual differences in neural patterns and intelligence. However, using univariate analyses, such as assessing neuronal activation levels, has provided an alternative approach.^81,82^ While univariate analyses have their advantages, such as simplicity and interpretability, they may not capture the complex patterns of neural activity that multivariate approaches can reveal. Secondly, our analysis was restricted to same-gender twin pairs from the Human Connectome Project dataset. In future research, accessing a more comprehensive twin dataset with a broader range of cognitive measures would allow us to represent the dynamics of twin relationships. Third, our study solely relies on resting-state fMRI data. While resting-state data offer valuable insights into intrinsic functional connectivity patterns, they may not fully capture brain activity during task performance or other cognitive states. Fourthly, we employed a K-fold cross-validation approach to obtain the association of intrinsic functional connectivity with the G-score. Despite the benefits of K-fold cross-validation, there is still a chance of high variance and overfitting for the limited dataset, contributing to added noise in the data. Nevertheless, in comparison to simpler validation techniques, the risk of overfitting is generally reduced with 10-fold cross-validation.^83^

In summary, the present study shows that functional connectivity patterns associated with general intelligence generalize across genetically identical twins but less across fraternal twins. These predictive effects were most pronounced in the default mode network, suggesting that higher-order association systems play a key role in supporting shared cognitive abilities. The contrast between identical and fraternal pairs indicates that greater genetic similarity enhances concordance in brain–behaviour associations. Although our design does not partition genetic and environmental influences, the results are consistent with genetic factors shaping connectivity patterns relevant to intelligence. By applying predictive modelling to twin data, this work offers a complementary perspective to classical twin approaches and provides new insights into the neural architecture supporting human intelligence.

## Supplementary Material

fcaf461_Supplementary_Data

## Data Availability

The neuroimaging data used in the present study are freely accessible and can be downloaded from the HCP database (https://humanconnectome.org/study/hcp-young-adult/). The behavioural data, excluding the restricted family data, are available for download from the HCP website (https://db.humanconnectome.org/). The software used in this study is freely accessible. The data analyses were performed using R and Python scripts, and the codes are available upon reasonable request. Codes used for this work can be accessed at https://github.com/arescobish/Twin-Study.

## References

[1] Spearman C . General intelligence, objectively determined and measured. The American Journal of Psychology. 1904;15(2):201.

[2] Gottfredson LS . Mainstream science on intelligence: An editorial with 52 signatories, history, and bibliography. Intelligence. 1997;24(1):13–23.

[3] Johnson W, Bouchard TJ, Krueger RF, McGue M, Gottesman II. Just one g: Consistent results from three test batteries. Intelligence. 2004;32(1):95–107.

[4] Jensen AR, Rushton JP. The g factor: The science of mental ability. 1998. Accessed 2 April 2024. http://arthurjensen.net/wp-content/uploads/2014/06/Review-of-Arthur-Robert-Jensens-The-g-Factor-The-Science-of-Mental-Ability-1998-by-John-Philippe-Rushton.pdf

[5] Johnson W, Te Nijenhuis J, Bouchard TJ. Still just 1 g: Consistent results from five test batteries. Intelligence. 2008;36(1):81–95.

[6] McFarland DJ . A single g factor is not necessary to simulate positive correlations between cognitive tests. J Clin Exp Neuropsychol. 2012;34(4):378–384. 22260190 10.1080/13803395.2011.645018

[7] Bartholomew DJ, Deary IJ, Lawn M. A new lease of life for Thomson’s bonds model of intelligence. Psychol Rev. 2009;116(3):567–579. 19618987 10.1037/a0016262

[8] Sternberg RJ . Beyond IQ: A Triarchic theory of human intelligence. 1985. Accessed 2 April 2024. https://books.google.com/books?hl=en&lr=&id=jmM7AAAAIAAJ&oi=fnd&pg=PR11&ots=atsA39BxpA&sig=07kWCfx-N8eyEoTzt1dEQ8JEuIE

[9] Horien C, Shen X, Scheinost D, Constable RT. The individual functional connectome is unique and stable over months to years. Neuroimage. 2019;189:676–687. 30721751 10.1016/j.neuroimage.2019.02.002 PMC6422733

[10] Jalbrzikowski M, Liu F, Foran W, et al Functional connectome fingerprinting accuracy in youths and adults is similar when examined on the same day and 1.5-years apart. Hum Brain Mapp. 2020;41(15):4187–4199. 32652852 10.1002/hbm.25118 PMC7502841

[11] Finn ES, Shen X, Scheinost D, et al Functional connectome fingerprinting: Identifying individuals using patterns of brain connectivity. Nat Neurosci. 2015;18(11):1664–1671. 26457551 10.1038/nn.4135 PMC5008686

[12] Ribeiro FL, Dos Santos FRC, Sato JR, Pinaya WHL, Biazoli CE. Inferring the heritability of large-scale functional networks with a multivariate ACE modeling approach. Network Neuroscience. 2021;5(2):527–548. 34189376 10.1162/netn_a_00189 PMC8233119

[13] Ge T, Holmes AJ, Buckner RL, Smoller JW, Sabuncu MR. Heritability analysis with repeat measurements and its application to resting-state functional connectivity. Proc Natl Acad Sci. 2017;114(21):5521–5526. 28484032 10.1073/pnas.1700765114 PMC5448225

[14] Teeuw J, Brouwer RM, Guimarães JPOFT, et al Genetic and environmental influences on functional connectivity within and between canonical cortical resting-state networks throughout adolescent development in boys and girls. Neuroimage. 2019;202:116073. 31386921 10.1016/j.neuroimage.2019.116073

[15] Cai H, Chen J, Liu S, Zhu J, Yu Y. Brain functional connectome-based prediction of individual decision impulsivity. Cortex. 2020;125:288–298. 32113043 10.1016/j.cortex.2020.01.022

[16] Shen X, Finn ES, Scheinost D, et al Using connectome-based predictive modeling to predict individual behavior from brain connectivity. Nat Protoc. 2017;12(3):506–518. 28182017 10.1038/nprot.2016.178 PMC5526681

[17] Yoo K, Rosenberg MD, Hsu WT, et al Connectome-based predictive modeling of attention: Comparing different functional connectivity features and prediction methods across datasets. Neuroimage. 2018;167:11–22. 29122720 10.1016/j.neuroimage.2017.11.010 PMC5845789

[18] Dubois J, Galdi P, Paul LK, Adolphs R. A distributed brain network predicts general intelligence from resting-state human neuroimaging data. Philos Trans R Soc Lond B Biol Sci. 2018;373(1756):20170284. 30104429 10.1098/rstb.2017.0284 PMC6107566

[19] Jiang R, Calhoun VD, Fan L, et al Gender differences in connectome-based predictions of individualized intelligence quotient and sub-domain scores. Cerebral Cortex. 2020;30(3):888–900. 31364696 10.1093/cercor/bhz134 PMC7132922

[20] Zhu J, Li Y, Fang Q, et al Dynamic functional connectome predicts individual working memory performance across diagnostic categories. Neuroimage Clin. 2021;30:102593. 33647810 10.1016/j.nicl.2021.102593 PMC7930367

[21] Beaty RE, Kenett YN, Christensen AP, et al Robust prediction of individual creative ability from brain functional connectivity. Proc Natl Acad Sci U S A. 2018;115(5):1087–1092. 29339474 10.1073/pnas.1713532115 PMC5798342

[22] Hearne LJ, Mattingley JB, Cocchi L. Functional brain networks related to individual differences in human intelligence at rest. Sci Rep. 2016;6:32328. 27561736 10.1038/srep32328 PMC4999800

[23] Thapaliya B, Akbas E, Chen J, et al Brain networks and intelligence: A graph neural network based approach to resting state fMRI data. 2023. Accessed 30 May 2024. https://arxiv.org/abs/2311.03520v2 10.1016/j.media.2024.103433 PMC11877132 39708510

[24] Berto S, Treacher AH, Caglayan E, et al Association between resting-state functional brain connectivity and gene expression is altered in autism spectrum disorder. Nat Commun. 2022;13(1):1–11. 35680911 10.1038/s41467-022-31053-5 PMC9184501

[25] Zhu D, Yuan T, Gao J, et al Correlation between cortical gene expression and resting-state functional network centrality in healthy young adults. Hum Brain Mapp. 2021;42(7):2236–2249. 33570215 10.1002/hbm.25362 PMC8046072

[26] Fu Y, Ma Z, Hamilton C, et al Genetic influences on resting-state functional networks: A twin study. Hum Brain Mapp. 2015;36(10):3959–3972. 26147340 10.1002/hbm.22890 PMC4704468

[27] Demeter DV, Engelhardt LE, Mallett R, et al Functional connectivity fingerprints at rest are similar across youths and adults and vary with genetic similarity. iScience. 2020;23(1):100801. 31958758 10.1016/j.isci.2019.100801 PMC6993008

[28] Miranda-Dominguez O, Feczko E, Grayson DS, Walum H, Nigg JT, Fair DA. Heritability of the human connectome: A connectotyping study. Netw Neurosci. 2018;2(2):175–199. 30215032 10.1162/netn_a_00029 PMC6130446

[29] Boomsma D, Busjahn A, Peltonen L. Classical twin studies and beyond. Nat Rev Genet. 2002;3(11):872–882. 12415317 10.1038/nrg932

[30] Craig JM, Calais-Ferreira L, Umstad MP, Buchwald D. The value of twins for health and medical research: A third of a century of progress. Twin Res Hum Genet. 2020;23(1):8–15. 31983355 10.1017/thg.2020.4

[31] Neale MC, Cardon LR. Methodology for genetic studies of twins and families. First Edition. Dordrecht: Springer; 1992: XXVI, 496. 10.1007/978-94-015-8018-2.

[32] Tang R, Etzel JA, Kizhner A, Braver TS. Frontoparietal pattern similarity analyses of cognitive control in monozygotic twins. Neuroimage. 2021;241:118415. 34298081 10.1016/j.neuroimage.2021.118415 PMC8958982

[33] Adhikari BM, Jahanshad N, Shukla D, et al Comparison of heritability estimates on resting state fMRI connectivity phenotypes using the ENIGMA analysis pipeline. Hum Brain Mapp. 2018;39(12):4893–4902. 30052318 10.1002/hbm.24331 PMC6218292

[34] Mollon J, Knowles EEM, Mathias SR, et al Genetic influence on cognitive development between childhood and adulthood. Mol Psychiatry. 2018;26(2):656–665. 30644433 10.1038/s41380-018-0277-0 PMC6570578

[35] Plomin R, Deary IJ. Genetics and intelligence differences: Five special findings. Mol Psychiatry. 2015;20(1):98. 25224258 10.1038/mp.2014.105 PMC4270739

[36] Quinn PD, López Pérez D, Kennedy DP, et al Visual search: Heritability and association with general intelligence. Genes Brain Behav. 2022;21(2):e12779. 35044053 10.1111/gbb.12779 PMC9744476

[37] Liu W, Kohn N, Fernández G. Intersubject similarity of personality is associated with intersubject similarity of brain connectivity patterns. Neuroimage. 2019;186:56–69. 30389630 10.1016/j.neuroimage.2018.10.062

[38] Elliott ML, Knodt AR, Cooke M, et al General functional connectivity: Shared features of resting-state and task fMRI drive reliable and heritable individual differences in functional brain networks. Neuroimage. 2019;189:516–532. 30708106 10.1016/j.neuroimage.2019.01.068 PMC6462481

[39] Yao C, Wang P, Xiao Y, et al Increased individual variability in functional connectivity of the default mode network and its genetic correlates in major depressive disorder. Sci Rep. 2025;15(1):1–12. 40087380 10.1038/s41598-025-92849-1 PMC11909136

[40] Jiang L, Genon S, Ye J, et al Gene transcription, neurotransmitter, and neurocognition signatures of brain structural-functional coupling variability. Nat Commun. 2025;16(1):1–18. 40817330 10.1038/s41467-025-63000-5 PMC12356869

[41] van Dijk KRA, Sabuncu MR, Buckner RL. The influence of head motion on intrinsic functional connectivity MRI. Neuroimage. 2012;59(1):431–438. 21810475 10.1016/j.neuroimage.2011.07.044 PMC3683830

[42] Van Essen DC, Smith SM, Barch DM, Behrens TEJ, Yacoub E, Ugurbil K. The WU-Minn Human Connectome Project: An overview. Neuroimage. 2013;80:62–79. 23684880 10.1016/j.neuroimage.2013.05.041 PMC3724347

[43] Gershon RC, Wagster MV, Hendrie HC, Fox NA, Cook KF, Nowinski CJ. NIH toolbox for assessment of neurological and behavioral function. Neurology. 2013;80(11 Suppl 3):S2–S6. 23479538 10.1212/WNL.0b013e3182872e5f PMC3662335

[44] Gur RC, Richard J, Hughett P, et al A cognitive neuroscience-based computerized battery for efficient measurement of individual differences: Standardization and initial construct validation. J Neurosci Methods. 2010;187(2):254–262. 19945485 10.1016/j.jneumeth.2009.11.017 PMC2832711

[45] Rosseel Y . Lavaan: An R package for structural equation modeling. J Stat Softw. 2012;48:1–36.

[46] Hu LT, Bentler PM. Cutoff criteria for fit indexes in covariance structure analysis: Conventional criteria versus new alternatives. Struct Equ Modeling. 1999;6(1):1–55.

[47] Tucker-Drob EM . Differentiation of cognitive abilities across the life span. Dev Psychol. 2009;45(4):1097–1118. 19586182 10.1037/a0015864 PMC2855504

[48] Feraco T, Cona G. Differentiation of general and specific abilities in intelligence. A bifactor study of age and gender differentiation in 8- to 19-year-olds. Intelligence. 2022;94:101669.

[49] Lynn R . Sex differences in intelligence: The developmental theory. Mankind Q. 2017;58(1):9–42.

[50] Glasser MF, Sotiropoulos SN, Wilson JA, et al The minimal preprocessing pipelines for the human connectome project. Neuroimage. 2013;80:105–124. 23668970 10.1016/j.neuroimage.2013.04.127 PMC3720813

[51] Smith SM, Beckmann CF, Andersson J, et al Resting-state fMRI in the human connectome project. Neuroimage. 2013;80:144–168. 23702415 10.1016/j.neuroimage.2013.05.039 PMC3720828

[52] Griffanti L, Salimi-Khorshidi G, Beckmann CF, et al ICA-based artefact removal and accelerated fMRI acquisition for improved resting state network imaging. Neuroimage. 2014;95:232–247. 24657355 10.1016/j.neuroimage.2014.03.034 PMC4154346

[53] Noble S, Spann MN, Tokoglu F, Shen X, Constable RT, Scheinost D. Influences on the test-retest reliability of functional connectivity MRI and its relationship with behavioral utility. Cereb Cortex. 2017;27(11):5415–5429. 28968754 10.1093/cercor/bhx230 PMC6248395

[54] Yan CG, Wang XD, Zuo XN, Zang YF. DPABI: Data processing & analysis for (resting-state) brain imaging. Neuroinformatics. 2016;14(3):339–351. 27075850 10.1007/s12021-016-9299-4

[55] Shen X, Tokoglu F, Papademetris X, Constable RT. Groupwise whole-brain parcellation from resting-state fMRI data for network node identification. Neuroimage. 2013;82:403–415. 23747961 10.1016/j.neuroimage.2013.05.081 PMC3759540

[56] Krishnan A, Williams LJ, McIntosh AR, Abdi H. Partial least squares (PLS) methods for neuroimaging: A tutorial and review. Neuroimage. 2011;56(2):455–475. 20656037 10.1016/j.neuroimage.2010.07.034

[57] McIntosh AR, Mišić B. Multivariate statistical analyses for neuroimaging data. Annu Rev Psychol. 2013;64:499–525. 22804773 10.1146/annurev-psych-113011-143804

[58] Diedenhofen B, Musch J. Cocor: A comprehensive solution for the statistical comparison of correlations. PLoS One. 2015;10(4):e0121945. 25835001 10.1371/journal.pone.0121945 PMC4383486

[59] Zou GY . Toward using confidence intervals to compare correlations. Psychol Methods. 2007;12(4):399–413. 18179351 10.1037/1082-989X.12.4.399

[60] Mo F, Zhao H, Li Y, et al Network localization of state and trait of auditory verbal hallucinations in schizophrenia. Schizophr Bull. 2024;50(6):1326–1336. 38401526 10.1093/schbul/sbae020 PMC11548935

[61] Cheng Y, Cai H, Liu S, et al Brain network localization of gray matter atrophy and neurocognitive and social cognitive dysfunction in schizophrenia. Biol Psychiatry. 2025;97(2):148–156. 39103010 10.1016/j.biopsych.2024.07.021

[62] Plomin R, Von Stumm S. The new genetics of intelligence. Nat Rev Genet. 2018;19(3):148. 29335645 10.1038/nrg.2017.104 PMC5985927

[63] Jiang S, Sun F, Yuan P, Jiang Y, Wan X. Distinct genetic and environmental origins of hierarchical cognitive abilities in adult humans. Cell Rep. 2024;43(4):114060. 38568809 10.1016/j.celrep.2024.114060

[64] de la Fuente J, Davies G, Grotzinger AD, Tucker-Drob EM, Deary IJ. A general dimension of genetic sharing across diverse cognitive traits inferred from molecular data. Nat Hum Behav. 2020;5(1):49–58. 32895543 10.1038/s41562-020-00936-2 PMC9346507

[65] He L, Liu W, Zhuang K, Meng J, Qiu J. Executive function-related functional connectomes predict intellectual abilities. Intelligence. 2021;85:101527.

[66] Brown SSG, Mak E, Clare I, et al Support vector machine learning and diffusion-derived structural networks predict amyloid quantity and cognition in adults with Down’s syndrome. Neurobiol Aging. 2022;115:112–121. 35418341 10.1016/j.neurobiolaging.2022.02.013 PMC10327571

[67] Arnatkeviciute A, Fulcher BD, Oldham S, et al Genetic influences on hub connectivity of the human connectome. Nat Commun. 2021;12(1):4237. 34244483 10.1038/s41467-021-24306-2 PMC8271018

[68] Cao H, Ingvar M, Hultman CM, Cannon T. Evidence for cerebello-thalamo-cortical hyperconnectivity as a heritable trait for schizophrenia. Transl Psychiatry. 2019;9(1):192. 31431615 10.1038/s41398-019-0531-5 PMC6702223

[69] Xin Y, Cui Y, Yu S, Liu N. Genetic contributions to brain criticality and its relationship with human cognitive functions. Proc Natl Acad Sci U S A. 2025;122(26):e2417010122. 40549906 10.1073/pnas.2417010122 PMC12232412

[70] Hill WD, Arslan RC, Xia C, et al Genomic analysis of family data reveals additional genetic effects on intelligence and personality. Mol Psychiatry. 2018;23(12):2347–2362. 29321673 10.1038/s41380-017-0005-1 PMC6294741

[71] Ma J, Xue K, Wang X, et al Gray matter volume abnormalities in vascular cognitive impairment and their association with gene expression profiles. Meta Radiol. 2023;1(3):100035.

[72] Kenett YN, Medaglia JD, Beaty RE, et al Driving the brain towards creativity and intelligence: A network control theory analysis. Neuropsychologia. 2018;118:79–90. 29307585 10.1016/j.neuropsychologia.2018.01.001 PMC6034981

[73] Ohtani T, Nestor PG, Bouix S, Saito Y, Hosokawa T, Kubicki M. Medial frontal white and gray matter contributions to general intelligence. PLoS One. 2014;9(12):e112691. 25551572 10.1371/journal.pone.0112691 PMC4281236

[74] Antonini A, Stryker MP. Rapid remodeling of axonal arbors in the visual cortex. Science. 1993;260(5115):1819–1821. 8511592 10.1126/science.8511592

[75] Gelfo F, Petrosini L. Environmental enrichment enhances cerebellar compensation and develops cerebellar reserve. Int J Environ Res Public Health. 2022;19(9):5697. 35565093 10.3390/ijerph19095697 PMC9099498

[76] Glahn DC, Winkler AM, Kochunov P, et al Genetic control over the resting brain. Proc Natl Acad Sci U S A. 2010;107(3):1223–1228. 20133824 10.1073/pnas.0909969107 PMC2824276

[77] Zhao B, Li T, Smith SM, et al Common variants contribute to intrinsic human brain functional networks. Nat Genet. 2022;54(4):508–517. 35393594 10.1038/s41588-022-01039-6 PMC11987081

[78] Smallwood J, Bernhardt BC, Leech R, Bzdok D, Jefferies E, Margulies DS. The default mode network in cognition: A topographical perspective. Nat Rev Neurosci. 2021;22(8):503–513. 34226715 10.1038/s41583-021-00474-4

[79] Fair DA, Cohen AL, Dosenbach NUF, et al The maturing architecture of the brain’s default network. Proc Natl Acad Sci U S A. 2008;105(10):4028–4032. 18322013 10.1073/pnas.0800376105 PMC2268790

[80] Menon V . 20 years of the default mode network: A review and synthesis. Neuron. 2023;111(16):2469–2487. 37167968 10.1016/j.neuron.2023.04.023 PMC10524518

[81] Weber S, Bühler J, Loukas S, et al Transient resting-state salience-limbic co-activation patterns in functional neurological disorders. Neuroimage Clin. 2024;41:103583. 38422831 10.1016/j.nicl.2024.103583 PMC10944183

[82] Shashidhara S, Spronkers FS, Erez Y. Individual-subject functional localization increases univariate activation but not multivariate pattern discriminability in the “multiple-demand” frontoparietal network. J Cogn Neurosci. 2020;32(7):1348–1368. 32108555 10.1162/jocn_a_01554 PMC7116248

[83] Arlot S, Celisse A. A survey of cross-validation procedures for model selection. Stat Surv. 2009;4:40–79.

