# Supplementary material for: Genetic contribution to intrinsic functional connectivity underlying general intelligence: evidence from adult twin study
Source: Brain Commun. 2025 Nov 21;7(6):fcaf461. doi: 10.1093/braincomms/fcaf461 (PMC12674170; doi:10.1093/braincomms/fcaf461)
Supplement: fcaf461_Supplementary_Data [file fcaf461_supplementary_data.pdf]

SUPPLEMENTARY RESULTS

Supplementary Table 1
Behavioral analysis of twins with G-score

	MZ Twins		DZ Twins	
	**Demographics (Mean ± SD)**	**Correlation with G-score**	**Demographics (Mean ± SD)**	**Correlation with G-score**
# of subjects	178		100	
Total Pairs	89		50	
Male/Female Pairs	38/51		20/30	
G-score	-0.05 ± 0.91		0.09 ± 0.91	
Age	29.21 ± 3.30	**-0.15***	29.23 ± 3.41	-0.13
Gender		**0.14***		**0.21***
Handedness		-0.03		-0.03
Adjusted G-score	-0.02 ± 0.89		0.10 ± 0.89	

Note: *p < 0.05; **p < 0.01, SD: Standard Deviation

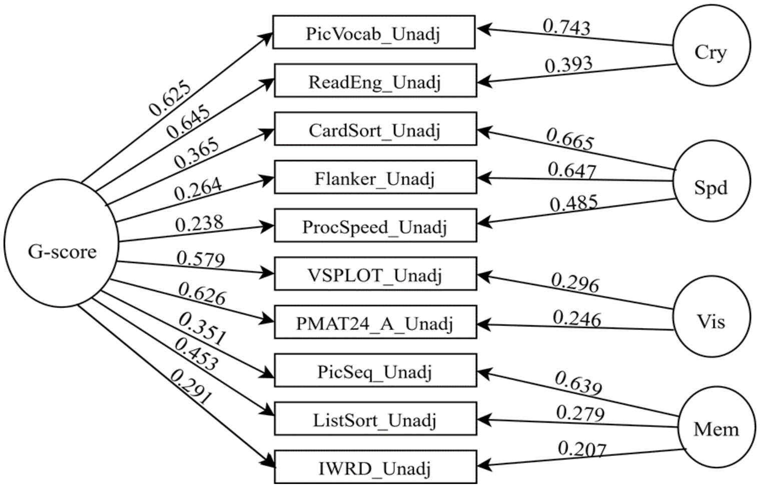

Supplementary Figure 1. Exploratory factor analysis of selected cognitive tasks for all subjects (Bi-factor analysis, N=278). Bi-factor model fit, including a G-score of intelligence (factor loading in all ten tasks) and four specialized factors (factor loadings on subsets of tasks). G-score: general intelligence, Cry: crystallized intelligence, spd: processing speed, vis: visuospatial ability, mem: memory.

Supplementary Table 2. Pearson Correlation between G-score and cognitive tasks within twins

	MZ Twins	DZ Twins
	**Correlation (r)**	**Correlation (r)**
Cognitive task scores		
PicVocab_Unadj	**0.65****	**0.61****
ReadEng_Unadj	**0.77****	**0.69****
PicSeq_Unadj	**0.42****	**0.31****
Flanker_Unadj	**0.22****	**0.32****
CardSort_Unadj	**0.40****	**0.35****
ProcSpeed_Unadj	**0.27****	**0.23****
PMAT24_A_CR	**0.75****	**0.76****
VSPLOT_TC	**0.70****	**0.72****
IWRD_TOT	**0.22****	**0.41****
ListSort_Unadj	**0.62****	**0.50****

Note: *p < 0.05; **p < 0.01

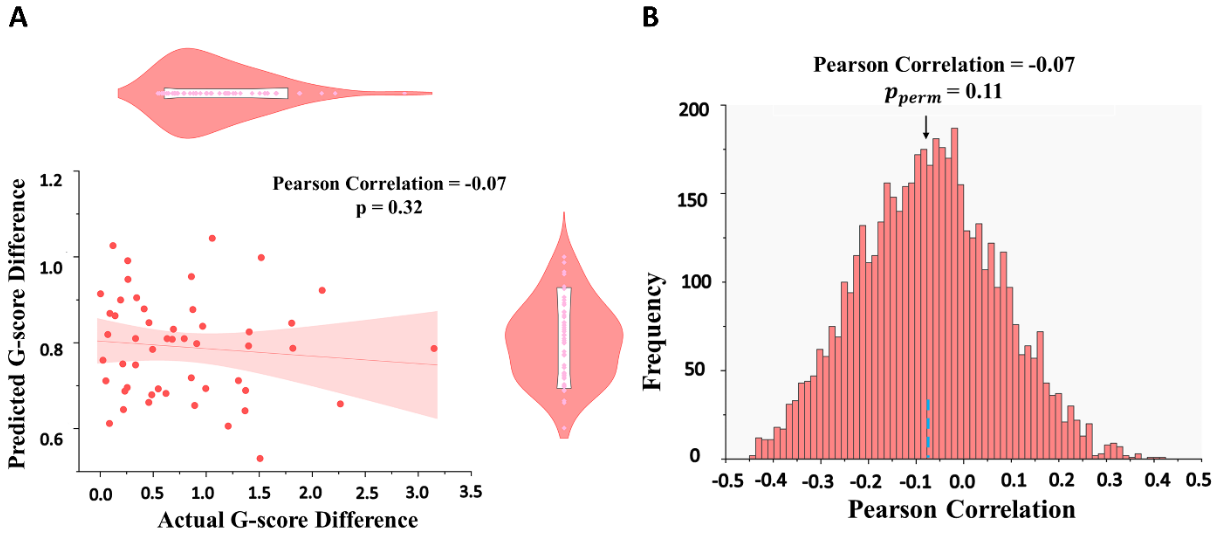

Supplementary Figure 2. Association of G-score difference with functional connectivity difference in DZ twins. (A) The accompanying violin plot, along with box plot, show the distribution of both actual and predicted scores (Pearson Correlation, r = -0.07, N = 50). (B) the histogram plot shows null distribution of G-score difference with a blue vertical line indicating the observed true score. The gray-shaded area refers to the distribution of DZ twins, which reveals a lack statistical significance (p: pvalue without permutation; p_perm: permutated pvalue).

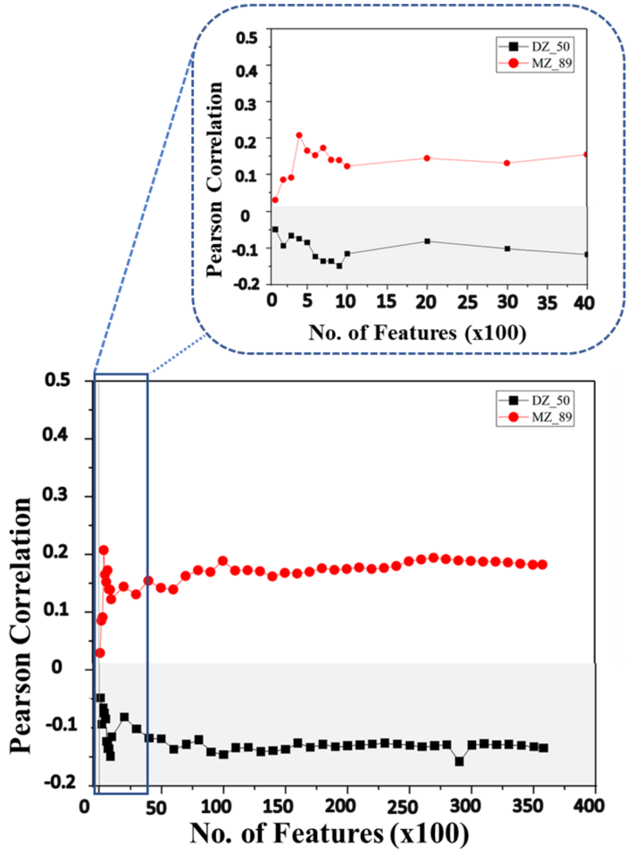

Supplementary Figure 3. Trend of correlation coefficient across varying feature numbers, that predicts G-score differences using functional connectivity differences within twin pairs. An optimal range of feature numbers was highlighted. The x-axis reflects the number of features selected, while the y-axis reflects correlation coefficient while predicting the G-score difference in twin pairs. The gray-shaded area showed a distribution that lacks statistical significance. DZ_50: Dizygotic twins (50 pairs), MZ_89: Monozygotic twins (89 pairs). (Each data point corresponds to the Pearson correlation coefficient calculated for a specific number of features).

Supplementary Table 3.
Brain regions that are most frequently selected across folds when predicting G-score difference

Node Name	Broadmann Areas	Network	MNI coordinates (X, Y, Z)	Connectivity Strength
Right Parietal Angular Gyrus	Angular Gyrus (BA39)	Fronto-Parietal	(47.8, -61.5, 34.7)	6
Right Inferior frontal Gyrus	ParsOrbitalis (BA47)	Medial-Frontal	(53.5, 24.8, 0.89)	6
Right Cerebellum	Cerebellum	Motor	(23.4, -59.3, -52.1)	4
Left Frontal Eye Fields	FEF (BA8)	Medial-frontal	(14.3, 36.9, 48.9)	4
Superior Temporal Gyrus	SupramargGyr (BA40)	Motor	(-42.2, -31.2, 15.8)	3
Right Brain Stem	BrainStem	Cerebellum	(9.6, -18.7, -30.6)	3
Right Basal Ganglia	Cerebellum	Basal-Ganglia	(35.5, -14.7, -18.4)	3
Left Inferior Temporal Gyrus	Inferior Temporal Gyrus (BA20)	Motor	(-37.8, -13.3, -29.6)	3

Supplementary Table 4.
Contribution of individual network when predicting G-score difference

	MZ Twins		DZ Twins		Corr Comparison
Network	Pearson Corr	Confidence Interval	Pearson Corr	Confidence Interval	Fisher's Z
Whole-brain	0.20	[0.02, 0.39]	-0.07	[-0.36, 0.16]	1.50
FrontoParietal	0.11	[0.03, 0.19]	-0.15	[-0.37, 0.07]	1.44
**DefaultMode**	**0.25***	**[0.1, 0.4]**	**-0.12**	**[-0.36, 0.12]**	**2.07**
MedialFrontal	-0.05	[-0.2, 0.1]	-0.07	[-0.32, 0.18]	0.11
Motor	0.21	[0.03, 0.39]	0.1	[-0.18, 0.2]	0.6
Vis-I	0.11	[-0.08, 0.3]	-0.2	[-0.38, -0.02]	1.72
Vis-II	-0.11	[-0.3, 0.08]	0.14	[-0.06, 0.34]	-1.38
VisAsso	-0.04	[-0.25, 0.17]	-0.04	[-0.22, 0.14]	0.00
BasalGanglia	0.15	[-0.07, 0.37]	-0.16	[-0.32, 0]	1.72
Limbic	0.12	[-0.06, 0.3]	-0.07	[-0.24, 0.1]	1.05
Cerebellum	-0.07	[-0.26, 0.12]	-0.12	[-0.28, 0.04]	0.27

Note: *p < 0.05; **p < 0.01

Supplementary Table 5
Comparison of prediction accuracy across different algorithms for predicting G-score using their co-twins functional connectivity

	MZ Twins		DZ Twins		Corr Comparison
**Algorithm**	**Pearson Corr**	**Confidence Interval**	**Pearson Corr**	**Confidence Interval**	**Fisher's Z**
**PLSR**	**0.35****	**[0.21, 0.45]**	**0.07**	**[-0.05, 0.18]**	**2.33**
**SVM**	**0.33****	**[0.17, 0.49]**	**0.05**	**[-0.05, 0.15]**	**2.31**
Decision Tree (DT)	0.26	[0.12, 0.4]	0.12	[-0.08, 0.36]	1.14
Logistic Regression (LR)	0.26	[0.14, 0.38]	0.11	[-0.06, 0.26]	1.22

Note: *p < 0.05; **p < 0.01; PLSR: Partial-least square regression; SVM: support vector machine

Supplementary Table 6
Comparison of prediction accuracy across different algorithms for predicting G-score difference using functional connectivity difference in twin pairs

	PLSR					SVR				
	MZ Twins		DZ Twins		Corr Comparison	MZ Twins		DZ Twins		Corr Comparison
**Network**	**Pearson Corr**	**Confidence Interval**	**Pearson Corr**	**Confidence Interval**	**Fisher's Z**	**Pearson Corr**	**Confidence Interval**	**Pearson Corr**	**Confidence Interval**	**Fisher's Z**
Whole-brain	0.20	[0.02, 0.39]	-0.07	[-0.36, 0.16]	1.50	0.19	[0.03, 0.35]	-0.01	[-0.13, 0.11]	1.11
FrontoParietal	0.11	[0.03, 0.19]	-0.15	[-0.37, 0.07]	1.44	0.15	[-0.05, 0.39]	-0.16	[-0.28, -0.04]	1.72
**DefaultMode**	**0.25***	**[0.1, 0.4]**	**-0.12**	**[-0.36, 0.12]**	**2.07**	**0.30***	**[0.19, 0.41]**	**-0.14**	**[-0.21, -0.07]**	**2.48**
MedialFrontal	-0.05	[-0.2, 0.1]	-0.07	[-0.32, 0.18]	0.11	**0.25***	**[0.05, 0.45]**	**-0.16**	**[-0.37. 0.06]**	**2.29**
Motor	0.21	[0.03, 0.39]	0.1	[-0.18, 0.2]	0.6	0.12	[-0.09. 0.33]	-0.14	[-0.35, 0.07]	1.44
Vis-I	0.11	[-0.08, 0.3]	-0.2	[-0.38, -0.02]	1.72	-0.04	[-0.21, 0.13]	-0.07	[-0.22, 0.08]	0.16
Vis-II	-0.11	[-0.3, 0.08]	0.14	[-0.06, 0.34]	-1.38	0.11	[0.06, 0.16]	-0.12	[-0.31, 0.07]	1.27
VisAsso	-0.04	[-0.25, 0.17]	-0.04	[-0.22, 0.14]	0.00	0.21	[0.03, 0.39]	0.09	[-0.11, 0.28]	0.6
BasalGanglia	0.15	[-0.07, 0.37]	-0.16	[-0.32, 0]	1.72	-0.13	[-0.32, 0.06]	0.12	[-0.05, 0.29]	-1.38
Limbic	0.12	[-0.06, 0.3]	-0.07	[-0.24, 0.1]	1.05	0.11	[-0.05. 0.26]	0.1	[-0.19, 0.23]	0.055
Cerebellum	-0.07	[-0.26, 0.12]	-0.12	[-0.28, 0.04]	0.27	0.12	[-0.08, 0.36]	0.11	[-0.08, 0.3]	0.059

Note: *p < 0.05; **p < 0.01; Corr: Pearson Correlation
